# Interleukin-15 is required for immunosurveillance and immunoprevention of HER2/neu-driven mammary carcinogenesis

**DOI:** 10.1186/s13058-015-0588-x

**Published:** 2015-05-22

**Authors:** Stefania Croci, Patrizia Nanni, Arianna Palladini, Giordano Nicoletti, Valentina Grosso, Giorgia Benegiamo, Lorena Landuzzi, Alessia Lamolinara, Marianna L. Ianzano, Dario Ranieri, Massimiliano Dall’Ora, Manuela Iezzi, Carla De Giovanni, Pier-Luigi Lollini

**Affiliations:** Laboratory of Immunology and Biology of Metastases, Department of Experimental, Diagnostic and Specialty Medicine, University of Bologna, Viale Filopanti 22, Bologna, 40126 Italy; Interdepartmental Centre for Cancer Research “Giorgio Prodi”, University of Bologna, Via Massarenti 9, Bologna, 40138 Italy; Laboratory of Experimental Oncology, Rizzoli Orthopedic Institute, Via di Barbiano 1/10, Bologna, 40136 Italy; CESI Aging Research Center, G. D’Annunzio University, Via Colle dell’Ara, Chieti Scalo, Chieti, 66013 Italy; Present address: Unit of Clinical Immunology, Allergy and Advanced Biotechnologies, Arcispedale Santa Maria Nuova-IRCCS, Viale Risorgimento 80, Reggio Emilia, 42123 Italy

## Abstract

**Introduction:**

We previously demonstrated that HER2/neu-driven mammary carcinogenesis can be prevented by an interleukin-12 (IL-12)-adjuvanted allogeneic HER2/neu-expressing cell vaccine. Since IL-12 can induce the release of interleukin-15 (IL-15), in the present study we investigated the role played by IL-15 in HER2/neu driven mammary carcinogenesis and in its immunoprevention.

**Methods:**

HER2/neu transgenic mice with homozygous knockout of IL-15 (here referred to as IL15KO/NeuT mice) were compared to IL-15 wild-type HER2/neu transgenic mice (NeuT) regarding mammary carcinogenesis, profile of peripheral blood lymphocytes and splenocytes and humoral and cellular responses induced by the vaccine.

**Results:**

IL15KO/NeuT mice showed a significantly earlier mammary cancer onset than NeuT mice, with median latency times of 16 and 20 weeks respectively, suggesting a role for IL-15 in cancer immunosurveillance. Natural killer (NK) and CD8+ lymphocytes were significantly lower in IL15KO/NeuT mice compared to mice with wild-type IL-15. The IL-12-adjuvanted allogeneic HER2/neu-expressing cell vaccine was still able to delay mammary cancer onset but efficacy in IL-15-lacking mice vanished earlier: all vaccinated IL15KO/NeuT mice developed tumors within 80 weeks of age (median latency of 53 weeks), whereas more than 70 % of vaccinated NeuT mice remained tumor-free up to 80 weeks of age. Vaccinated IL15KO/NeuT mice showed less necrotic tumors with fewer CD3+ lymphocyes and lacked perforin-positive infiltrating cells compared to NeuT mice. Concerning the anti-vaccine antibody response, antibody titer was unaffected by the lack of IL-15, but less antibodies of IgM and IgG1 isotypes were found in IL15KO/NeuT mice. A lower induction by vaccine of systemic interferon-gamma (IFN-γ) and interleukin-5 (IL-5) was also observed in IL15KO/NeuT mice when compared to NeuT mice. Finally, we found a lower level of CD8+ memory cells in the peripheral blood of vaccinated IL15KO/NeuT mice compared to NeuT mice.

**Conclusions:**

We demonstrated that IL-15 has a role in mammary cancer immunosurveillance and that IL-15-regulated NK and CD8+ memory cells play a role in long-lasting immunoprevention, further supporting the potential use of IL-15 as adjuvant in immunological strategies against tumors.

**Electronic supplementary material:**

The online version of this article (doi:10.1186/s13058-015-0588-x) contains supplementary material, which is available to authorized users.

## Introduction

Activation of the immune system to prevent onset and progression of tumors not caused by infective agents is emerging as a feasible perspective. Targeted immunoprevention was in fact obtained in mouse models, the most studied of which consists in HER2/neu-transgenic mice [1]. Vaccination of BALB/c mice transgenic for rat HER2/neu (NeuT mice) with an interleukin-12 (IL-12)-adjuvanted allogeneic HER2/neu-expressing cell vaccine gave an effective and long-lasting prevention of mammary carcinogenesis, provided that vaccinations started at the preneoplastic stage and were repeated cyclically for the mouse’s lifetime [2, 3]. Identification of immune mechanisms at the basis of vaccine efficacy is important to move toward clinical application and to optimize the vaccine (e.g., with a choice of new adjuvants).

Recently, it has been reported that IL-12 can induce a rapid release of interleukin-15 (IL-15) by tumor-associated and tumor-infiltrating macrophages [4, 5]. Such induction is transient but is necessary to favor infiltration of tumors by leukocytes and for the antitumor and antimetastatic effects exerted by IL-12 [5]. In addition interferon-γ (IFN-γ), the main mediator of the activities of IL-12, can also induce IL-15 [6]. Thus we hypothesized that the IL-12-adjuvanted cell vaccine might induce IL-15, which in turn might have a role in cancer immunoprevention.

IL-15 belongs to the four α-helix bundle cytokine family and has some overlapping activities with interleukin-2 (IL-2). It signals through a heterotrimeric receptor complex composed of the shared IL-2/15Rβ (CD122) and common γ chain (γC) and a specific α subunit (IL-15Rα). IL-15 is necessary for the development and function of CD8+ T lymphocytes, natural killer (NK) cells, invariant NKT cells and a subset of intestinal intraepithelial lymphocytes [7, 8]. IL-15 can exert antitumor and antimetastatic activities [9–11] and IL-12 and IL-15 can act synergistically to induce antitumor immune responses [12]. Due to its promising antitumor activities, IL-15 is currently being evaluated in some clinical trials for advanced and metastatic tumors [13].

Here we studied the role played by IL-15 in HER2/neu-driven mammary carcinogenesis and immunoprevention, through mice knocked out for the IL-15 gene and transgenic for the HER2/neu oncogene. Mammary carcinogenesis and efficacy of cancer immunoprevention, and immune mechanisms, were studied in IL15-deficient and IL15-proficient NeuT mice.

## Methods

### Mice

Mice knocked out for the IL-15 gene and transgenic for the transforming activated rat HER2/neu oncogene driven by the mouse mammary tumor virus promoter, on a BALB/c background (throughout referred to as IL15KO/NeuT mice) were kindly provided by Dr. Silvia Bulfone Paus (Faculty of Human and Medical Sciences, University of Manchester, Manchester, UK and Department of Immunology and Cell Biology, Research Center Borstel, Borstel, Germany). NeuT mice and IL15KO/NeuT mice were bred in the local animal facility. Animal experiments were authorized by the Ethical Scientific Committee for Animal Experimentation of the University of Bologna (Bologna, Italy). Mammary pads were inspected weekly: masses with a mean diameter exceeding 3 mm were considered tumors. Mice were sacrificed when tumors were present in all mammary glands or when a single mass exceeded a mean diameter of 1.5 cm.

### Vaccination

Mice were vaccinated lifelong with allogeneic (H-2^q^) murine mammary carcinoma cells expressing high levels of HER2/neu and transduced with IL-12 genes [3], starting at the sixth week of age. Vaccine cells were proliferation-blocked by treatment with mitomycin C (40 μg/ml Sigma-Aldrich, Milan, Italy) and administered intraperitoneally in 0.4 ml of phosphate-buffered saline (PBS) (Invitrogen, Milan, Italy). Control mice received PBS alone. The vaccination schedule was reported previously: twice weekly for 2 weeks, followed by 2 weeks of rest [3]. Mice were monitored weekly for tumor occurrence. Tumor progression of control mice was equal to that of untreated mice.

### Immunophenotyping

Blood was collected in EDTA tubes (BD Pharmingen, San Diego, CA, USA) and leukocytes counted in a Neubauer hemocytometer using Turk’s solution (Merck KGaA, Darmstadt, Germany). Lymphocytes and granulocytes were distinguished by morphology of nuclei. After lysis of red blood cells, FcγII and FcγIII receptors were blocked with anti-CD16/CD32 antibodies at 5 μg/ml (Mouse BD Fc Block™, BD Pharmingen) then specific primary antibodies were added: fluorescein isothiocyanate (FITC)-conjugated rat anti-mouse CD45R/B220 monoclonal antibody; phycoerythrin (PE)-conjugated hamster anti-mouse CD3e monoclonal antibody; PE-conjugated rat anti-mouse CD335 (NKp46) monoclonal antibody; FITC-conjugated rat anti-mouse CD49b (clone DX5) monoclonal antibody; FITC-conjugated rat anti-mouse CD8a monoclonal antibody; PE-conjugated rat anti-mouse CD4 monoclonal antibody; PE-conjugated rat anti-mouse CD44 monoclonal antibody. All antibodies were from BD Pharmingen. The Partec CyFlow**®** space cytofluorimeter was used and analysis was performed with Windows™ FloMax® software. Through backgating from the CD3+ and B220+ clusters, we selected the lymphocyte gate in the FSC-SSC dot plot. At least 10,000 events were acquired in the gate. The analysis template was kept constant in the various experiments. The same reagents and procedures were used for splenocyte immunophenotyping.

### RNA extraction and real-time PCR

RNA was extracted from frozen mammary glands with Trizol reagent (Life Technologies, Milan, Italy). Rat HER2/neu expression was quantified by real-time PCR as previously reported [14]. NKp46, CD8 and CD4 expression was determined by real-time PCR using the PrimePCR SYBR Green Assays (qMmuCID0015089, qMmuCID0016523, qMmuCID0022320 respectively, Bio-Rad, Milan, Italy) following the instruction manual. GAPDH was used as the housekeeping gene (qMmuCED0027497, Bio-Rad).

### Quantification of cytokines

Levels of cytokines in plasma were measured using the Bio-Plex™ Mouse Cytokine 23-Plex bead assay (Bio-Rad) following the manufacturer’s instructions. Production of IFN-γ in culture supernatants was quantified by enzyme-linked immunosorbent assay (ELISA) (Quantikine Mouse IFN-γ, R&D Systems, Minneapolis, MN, USA). Levels of the bioactive IL-15 were measured in serum with the Mouse IL-15/IL-15R Complex ELISA Ready-SET-Go (eBioscience, San Diego, CA, USA). We set up a standard curve of nine points by twofold serial dilutions of the top standard and we performed an overnight incubation at 4 °C of the samples to reach the maximal sensitivity. Serum samples were diluted 20 times with assay buffer.

### Mixed lymphocyte-tumor cell cultures

Mononuclear cells were obtained from spleens using cell strainers with 40 μm pores (Falcon, Oxnard, CA, USA). After lysis of red blood cells, splenocytes were co-cultured at a 10:1 ratio with proliferation-blocked mammary carcinoma cells for 6 days in RPMI 1640 supplemented with 10 % fetal bovine serum and with 10 units/ml of recombinant IL-2 (Peprotech, Rocky Hill, NJ, USA). Splenocytes yields were determined by counting with a Neubauer’s hemocytometer (viable and dead cells were counted separately using the erythrosine dye exclusion method).

### Antibody response

Mice were bled from the ventral caudal vein and sera were stored frozen at –80 °C. To determine the level of anti-vaccine antibodies, sera were used in an indirect immunofluorescence assay to stain viable vaccine cells followed by cytofluorometric analysis as previously described [3]. Sera were diluted 1:65. To detect total anti-vaccine immunoglobulin (Ig)Gs, F(ab’)2 fragments of goat anti-mouse IgGs (H+L) labeled with Alexa Fluor® 488 (20 μg/ml, Life Technologies) were used as secondary antibodies. To detect specific antibody isotypes FITC-conjugated rat anti-mouse IgM, IgG1, IgG2a, IgG2b, IgG3 (50 μg/ml, BD Pharmingen™) were used. The intensity of fluorescence of each serum sample was normalized to the expression of rat HER2/neu by the target cells (determined using the monoclonal antibody Ab4, clone 7.16.4, 5 μg/ml, Oncogene Research Products, Cambridge, MA, USA).

### Morphologic and immunohistochemical analyses

Whole mounts were prepared as previously described [15]. Mammary tumors were fixed in formalin and embedded in paraffin. Hematoxylin and eosin staining (H&E) was used to assess histology. Infiltration by CD3+ and perforin-positive cells was determined through immunohistochemistry with anti-CD3 (Ab828; Abcam, Cambridge, UK) and anti-perforin (Ab16074, Abcam) antibodies as previously described [16]. Immunostaining was developed using alkaline phosphatase-conjugated streptavidin (Thermo Fisher Scientific, Waltham, MA, USA) and vulcan fast red chromogen (Biocare Medical, Concord, CA, USA). Slides were counterstained with hematoxylin (BioOptica, Milan, Italy) and images were acquired by Leica DMRD optical microscope (Leica, Milton Keynes, UK).

### Statistical analysis

Differences in tumor-free survival curves were analyzed by the Mantel-Haenszel test. Cytokine, cell frequency, real-time PCR data and antibody levels were compared by the Student’s *t* test or nonparametric Wilcoxon test.

## Results

### Lack of IL-15 hastens HER2/neu-driven mammary carcinogenesis

**Fig. 1:**
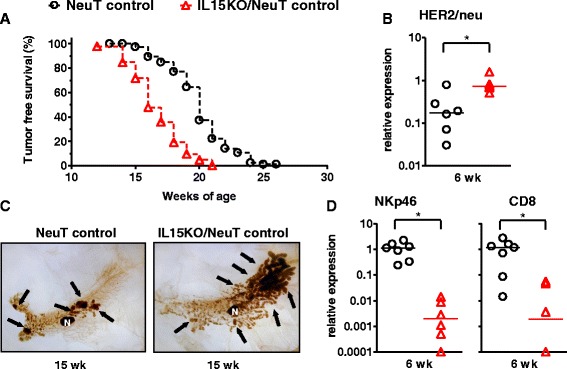
Earlier mammary carcinogenesis in IL15KO/NeuT mice. **a** Tumor-free survival curves (IL15KO/NeuT mice versus NeuT mice, *p* <0.01 by the Mantel-Haenszel test). IL15KO/NeuT mice: *n* = 46; NeuT mice: *n* = 117. **b** Expression of rat HER2/neu transcript in preneoplastic mammary glands from 6-week-old mice. Expression was evaluated by real-time PCR applying the ΔΔCt method using GAPDH as the housekeeping gene and the HER2/neu-positive TUBO mammary carcinoma cell line as reference. **c** Whole mounts of mammary glands obtained from 15-week-old mice. N = lymph node. **d** Expression of NKp46 and CD8 mRNAs in preneoplastic mammary glands from 6-week-old mice. Expression was evaluated by real-time PCR applying the ΔΔCt method using GAPDH as the housekeeping gene and mammary glands from 6-week-old BALB/c mice as reference. ^*^ = *p* <0.05 at Student’s *t* test. *IL15KO/NeuT* BALB/c mice transgenic for rat HER2/neu oncogene and knocked out for IL-15, *NeuT* BALB/c mice transgenic for rat HER2/neu oncogene

Mice knocked out for IL-15 and transgenic for HER2/neu oncogene (IL15KO/NeuT mice) developed mammary carcinomas with a median latency of 16 weeks, significantly shorter than the median latency of 20 weeks displayed by HER2/neu transgenic mice with wild-type IL-15 (NeuT mice) (Fig. 1a). Mammary carcinogenesis was thus earlier in the absence of IL-15 indicating a role in immunosurveillance for this cytokine. To confirm at the molecular level that the absence of IL-15 induced an earlier mammary carcinogenesis, we evaluated the expression of HER2/neu oncogene in mammary glands from 6-week-old mice. HER2/neu is the driving force of carcinogenesis in this tumor model, thus its expression can be considered as a molecular marker of tumor progression. IL15KO/NeuT mice showed a higher expression of HER2/neu transgene in preneoplastic mammary tissue compared to IL-15 wild-type NeuT mice (Fig. 1b). This data is consistent with the greater number of preneoplastic/neoplastic cells in mammary glands of IL15KO/NeuT mice, compared to NeuT mice, observed in whole mounts of inguinal mammary glands from 15-week-old mice (Fig. 1c), and confirms the more precocious onset of preneoplastic/neoplastic HER2/neu-positive cells.

### Lack of IL-15 impairs the efficacy of cancer preventive vaccine

**Fig. 2:**
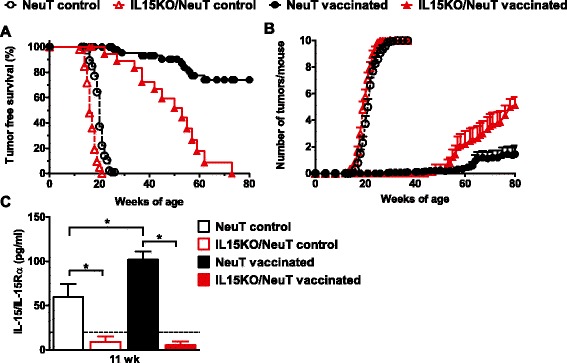
Impaired immunoprevention of mammary carcinogenesis in IL15KO/NeuT mice. Vaccinated mice received a lifelong IL-12-adjuvanted allogeneic HER2/neu-expressing cell vaccine, as described in “Material and methods”. Control mice received PBS. Control mice are the same reported in Fig. 1 and are shown here for comparison. **a** Tumor-free survival curves (IL15KO/NeuT mice versus NeuT mice, *p* <0.01 by the Mantel-Haenszel test). Vaccinated IL15KO/NeuT mice: n = 24; vaccinated NeuT mice: n = 56. **b** Tumor multiplicity is shown as mean ± SEM. **c** Serum levels of the bioactive IL-15/IL-15Rα determined 20 hours after vaccination in mice of 11 weeks of age by ELISA. The dotted line indicates the sensitivity of the assay. *ELISA* enzyme-linked immunosorbent assay, *IL-15* interleukin 15, *IL15KO/NeuT* BALB/c mice transgenic for rat HER2/neu oncogene and knocked out for IL-15, *NeuT* BALB/c mice transgenic for rat HER2/neu oncogene, *PBS* phosphate-buffered saline, *SEM* standard error of the mean

In NeuT mice an IL-12-adjuvanted allogeneic HER2/neu-expressing cell vaccine, repeatedly administered, was found to delay mammary cancer onset, leading to a more than doubled mice lifetime expectancy [2, 3]. We tested the efficacy of this vaccine in the IL15KO/NeuT mice. The preventive vaccine effectively delayed mammary carcinogenesis also in IL15KO/NeuT mice but immunoprevention did not last as long as in NeuT mice. At 80 weeks of age all IL15KO/NeuT mice had developed tumors, while about 70 % of NeuT mice were still free from mammary carcinomas (*p* <0.01 between the tumor-free survival curves) (Fig. 2a). Moreover, the mean number of tumors per mouse was higher in IL15KO/NeuT mice at sacrifice compared to NeuT mice (Fig. 2b) further sustaining a role for IL-15 in immunoprevention. There is increasing evidence that IL-15 is only secreted in complex with its unique receptor, IL-15Rα [17]. Vaccinated NeuT mice showed higher levels of IL-15/IL-15Rα heterodimers in serum than untreated NeuT mice (Fig. 2c) indicating that the IL-12-expressing vaccine can induce IL-15 in its bioactive form. Instead, IL15KO/NeuT mice had levels of the IL-15/IL-15Rα complex lower than the limit of detection of the ELISA (Fig. 2c).

### Immunological pathways regulated by IL-15

The lack of IL-15 caused an earlier mammary carcinogenesis and an earlier loss of efficacy of the preventive vaccine. To identify the immune mechanisms involved in these phenomena, we immunophenotyped peripheral blood lymphocytes and splenocytes from NeuT versus IL15KO/NeuT control and vaccinated mice. The kinetic study of humoral and cellular responses elicited by vaccine was performed at several time points during vaccination cycles, after one, two and three vaccination cycles in comparison with age-matched controls (7, 11 and 15 weeks of age respectively) and after six and ten vaccination cycles (29 and 42 weeks of age) only in vaccinated mice, since at these time points untreated mice developed tumors and had to be sacrificed.

**Fig. 3:**
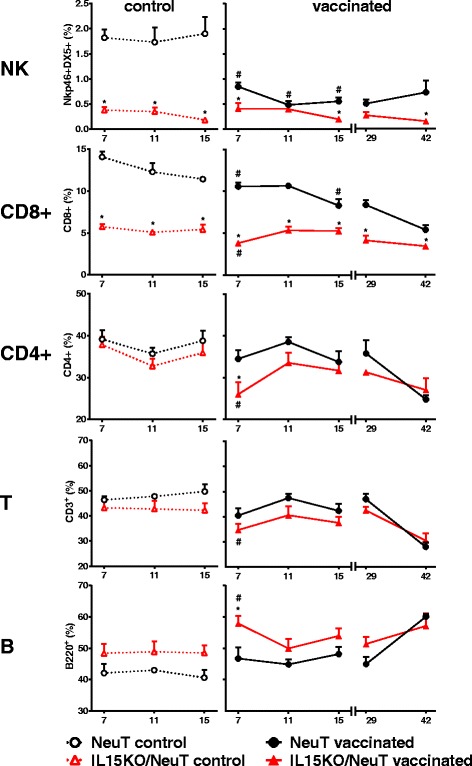
Relative frequency of peripheral blood lymphocyte subsets in IL-15-deficient and IL-15-proficient, control and vaccinated NeuT mice. Lymphocyte subsets were analyzed by flow cytometry in control and vaccinated mice 20 hours after vaccination. A lymphocyte gate was defined in the FSC-SSC dot plot then the percentage of B220+, CD3+, CD4+, CD8+ and Nkp46 and DX5 double-positive cells was determined. Significance of comparisons (Student’s *t* test): ^*^ *p* <0.05, IL15KO/NeuT versus NeuT mice; ^#^ *p* <0.05, vaccinated versus control mice within the same strain. Mean ± SEM is shown (three to five mice per group). *IL15KO/NeuT* BALB/c mice transgenic for rat HER2/neu oncogene and knocked out for IL-15, *NeuT* BALB/c mice transgenic for rat HER2/neu oncogene, *SEM* standard error of the mean

**Fig. 4:**
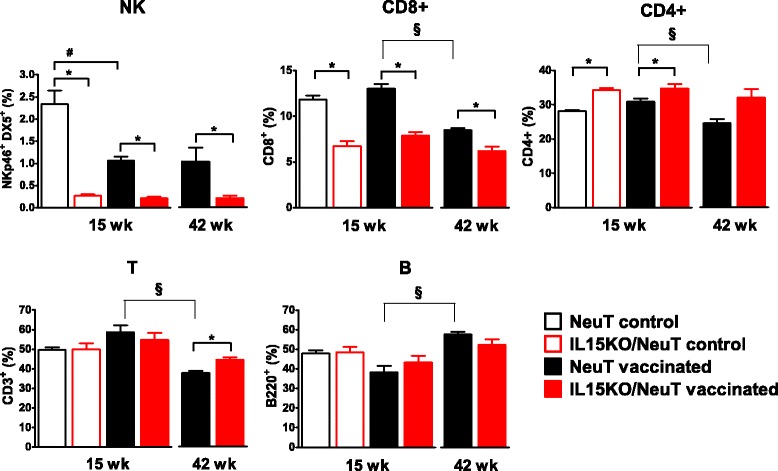
Relative frequency of spleen lymphocyte subsets in IL-15-deficient and IL-15-proficient, control and vaccinated NeuT mice. Splenocytes were analyzed by flow cytometry in control and vaccinated mice. The percentage of B220+, CD3+, CD4+, CD8+ and Nkp46 and DX5 double-positive cells is reported. Significance of comparisons (Student’s *t* test): ^*^ *p* <0.05, IL15KO/NeuT versus NeuT mice; ^#^ *p* <0.05, vaccinated versus control mice within the same strain; ^§^ *p* <0.05, 15- versus 42-week-old mice within the same strain. Mean ± SEM is shown (three to six mice per group). *IL15KO/NeuT* BALB/c mice transgenic for rat HER2/neu oncogene and knocked out for IL-15, *NeuT* BALB/c mice transgenic for rat HER2/neu oncogene, *SEM* standard error of the mean

In naive mice, IL-15 deficiency did not modify the level of circulating leukocytes (see Additional file 1) but had a significant impact on lymphocyte subsets. IL15KO/NeuT mice had lower percentage (Fig. 3) and absolute number (see Additional file 2) of NK and CD8+ cells in peripheral blood. Spleen cell yield from IL15KO/NeuT mice was lower than that of NeuT mice (see Additional file 3, 15-week-old control mice) and frequency (Fig. 4) as well as absolute number (see Additional file 3) of NK and CD8+ spleen lymphocytes were significantly decreased in IL-15-lacking mice. In addition, IL15KO/NeuT mice showed 1000-fold lower expression of NKp46 and CD8 genes in preneoplastic mammary glands compared to NeuT mice (Fig. 1d).

Vaccination of IL-15-proficient NeuT mice caused a significant and persistent decrease in the percentage and in the absolute number of NK cells in peripheral blood and in spleen (compare control and vaccinated NeuT mice in Figs. 3 and 4 and see Additional files 2 and 3). Moreover, at 42 weeks of age vaccinated NeuT mice showed a significant decrease in circulating lymphocyte concentration (see Additional file 1) and in spleen yield (see Additional file 3) with a parallel increase in the percentage of B lymphocytes and a decrease in the percentage of CD3+, CD4+, CD8+ T lymphocytes (Figs. 3 and 4, comparison: 15- versus 42-week-old NeuT mice). Therefore the number of T cells decreased more than that of B and NK cells during the time course of vaccination.

In IL15KO/NeuT mice vaccination induced transient alterations of circulating populations: higher B lymphocyte and lower CD4+ and CD8+ T lymphocyte percentages were observed in comparison to both IL15KO/NeuT controls and to vaccinated NeuT mice after the first vaccination cycle (7-week-old mice) (Fig. 3). Lifelong vaccination did not modify NK and CD8+ levels of IL15KO/NeuT mice, which remained significantly lower than in vaccinated NeuT mice up to 42 weeks (Figs. 3 and 4).

**Fig. 5:**
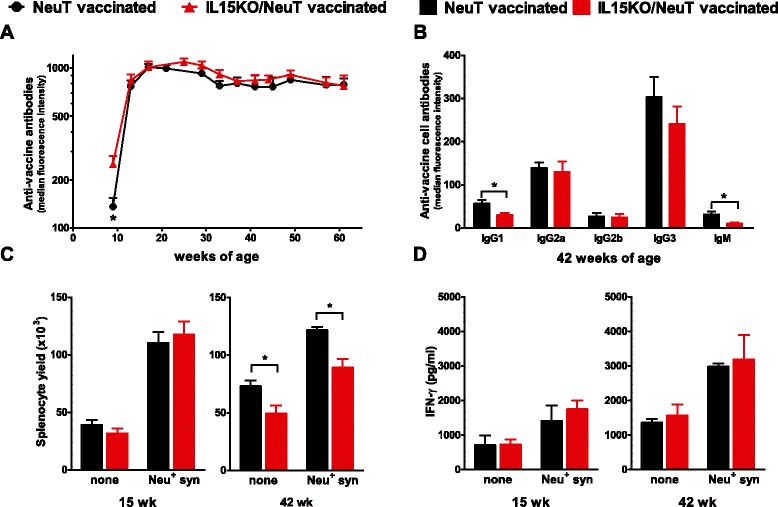
Immune mechanisms induced by the preventive vaccine in IL15KO/NeuT and NeuT mice. **a** Anti-vaccine antibodies. Sera from vaccinated mice were tested against vaccine cells by indirect immunofluorescence followed by cytofluorometric analysis. **b** Anti-vaccine cell antibody isotypes. Mean ± SEM is shown (eight mice per group). **c** Yield of splenocyte cultures from 15- and 42-week-old vaccinated mice (IL15KO/NeuT versus NeuT) restimulated by the 6-day co-culture with HER2/neu-expressing syngeneic cells. **d** IFN-γ production by splenocytes restimulated as in **c**. Mean ± SEM is shown (three to six mice per group). ^*^ *p* <0.05 by the Student’s *t* test. *IFN-γ* interferon-gamma, *IL15KO/NeuT* BALB/c mice transgenic for rat HER2/neu oncogene and knocked out for IL-15, *NeuT* BALB/c mice transgenic for rat HER2/neu oncogene, *SEM* standard error of the mean

Proven mechanisms at the basis of the efficacy of the preventive vaccine in NeuT mice were the induction of a fast and sustained antibody response against the HER2/neu oncoantigen and the release of IFN-γ [2, 3, 15, 18]. In IL15KO/NeuT mice the vaccine induced a faster antibody response after the first vaccination cycle, then antibodies reached plateau levels similar to those of NeuT mice (Fig. 5a). However, differences in the antibody isotypes were found. Specifically less IgM and IgG1 were detected in IL15KO/NeuT mice (Fig. 5b).

To analyze the activation of cell-mediated immunity, we co-cultured splenocytes from vaccinated mice with syngeneic neu-expressing cells and we determined lymphocyte yield and production of IFN-γ, the main mediator of the activities of IL-12, after 6 days of co-cultures. The presence of HER2/neu antigen effectively stimulated the splenocytes: about a twofold increase in cell yield and in IFN-γ production was observed following co-culture in both IL-15-deficient and IL-15-proficient NeuT mice. Yield of splenocyte cultures from IL-15 knockout mice was equal to that of splenocytes cultures from wild-type mice at 15 weeks of age but at later time points (42 weeks of age) it was lower (Fig. 5c). Nevertheless concentration of IFN-γ in supernatants was comparable between the two mouse strains both at 15 and 42 weeks of age (Fig. 5d).

**Fig. 6:**
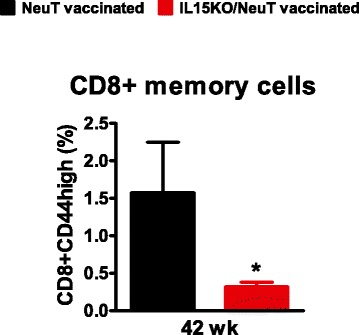
Memory CD8+ T cells at late phases of vaccination. Memory CD8+ T cells were defined as CD44high by flow cytometry. Percentage of CD8+CD44high lymphocytes is reported. ^*^ *p* <0.05 by the Student’s *t* test

Vaccinated IL-15-deficient NeuT mice had less memory CD8+ lymphocytes in peripheral blood compared to vaccinated IL-15 wild-type NeuT mice (Fig. 6).

The concentration of a panel of cytokines in plasma from IL-15-deficient and IL-15-proficient NeuT mice 20 hours after vaccine administration was studied and compared to those of corresponding untreated controls (see Additional file 4). A rise in IFN-γ and IL-5 was induced by the vaccine only in IL-15 wild-type NeuT mice. Several cytokines were similarly modified by the vaccine in IL-15-deficient and IL-15-proficient NeuT mice, with a significant rise in interleukin-1 alpha (IL-1α), regulated on activation, normal T cell expressed and secreted (RANTES), interleukin-13 (IL-13), monocyte chemoattractant protein-1 (MCP-1) and macrophage inflammatory protein-1 alpha (MIP-1α) and a decrease in granulocyte macrophage-colony stimulating factor (GM-CSF). Untreated IL-15-deficient NeuT mice showed higher levels of granulocyte colony-stimulating factor (G-CSF) and keratinocyte-derived chemokine (KC) and lower levels of RANTES and interleukin-17 (IL-17) than IL-15-proficient NeuT mice. In IL-15KO/NeuT mice the vaccine induced a rise in RANTES, IL-17 and KC reaching levels similar to those of IL-15 wild-type NeuT mice (see Additional file 4).

### Recruitment of immune cells in mammary tissue by vaccination

**Fig. 7:**
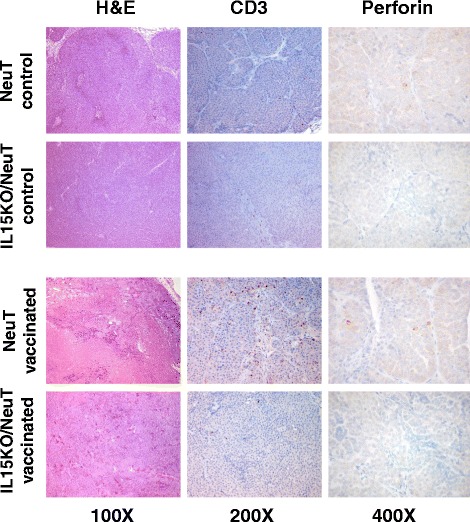
Morphology and infiltration by CD3+ and perforin + lymphocytes in tumors of control and vaccinated IL15KO/NeuT and NeuT mice. Morphology was evaluated by staining with H&E. Presence of CD3+ and perforin-producing cells was evaluated by IHC with a chromogenic reaction. *H&E* hematoxylin and eosin, *IHC* immunohistochemistry, *IL15KO/NeuT* BALB/c mice transgenic for rat HER2/neu oncogene and knocked out for IL-15, *NeuT* BALB/c mice transgenic for rat HER2/neu oncogene

**Fig. 8:**
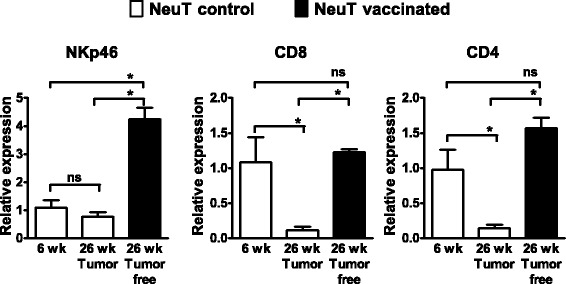
Infiltrating lymphocytes in mammary glands and tumors from NeuT mice. Levels of NKp46, CD8 and CD4 transcripts were quantified by real-time PCR in preneoplastic mammary tissue from 6-week-old untreated NeuT mice, tumors from 26-week-old untreated NeuT mice and tumor-free mammary glands from 26-week-old vaccinated NeuT mice. The ΔΔCt method was applied using GAPDH as the housekeeping gene and mammary glands from 6-week-old BALB/c mice as reference. ^*^ = *p* <0.05 at Student’s *t* test, ns = not significant. *IL15KO/NeuT* BALB/c mice transgenic for rat HER2/neu oncogene and knocked out for IL-15, *NeuT* BALB/c mice transgenic for rat HER2/neu oncogene

Tumors from vaccinated versus untreated IL-15-deficient and IL-15-proficient NeuT mice were analyzed for morphology and infiltrating CD3+ and perforin-positive cells. Tumors from vaccinated NeuT mice showed several necrotic areas, which were rarely present in tumors from vaccinated IL15KO/NeuT mice as well as in untreated mice (Fig. 7, H&E). Vaccination induced in NeuT mice a high level of infiltrated CD3+ lymphocytes within the tumor mass and in intra- and peritumoral stroma (Fig. 7), which was not elicited in IL15KO/NeuT mice. The few CD3+ lymphocytes infiltrating IL15KO/NeuT tumors were found mainly within the tumor stroma (Fig. 7). Tumors from NeuT mice showed few perforin-positive cells, slightly more numerous in tumors from vaccinated mice. Instead, tumors from IL15KO/NeuT mice (both untreated and vaccinated) did not show any perforin-positive cells (Fig. 7). To determine whether the vaccine could induce the recruitment of immune cells at the site of carcinogenesis, we investigated by real-time PCR the expression of NKp46, CD8 and CD4 mRNAs in tumor-free mammary glands from 26-week-old vaccinated NeuT mice compared to that in preneoplastic mammary glands from untreated NeuT mice at 6 weeks of age, at the baseline of vaccination. NKp46 levels were significantly higher in mammary glands from vaccinated mice, whereas CD8 and CD4 levels were comparable (Fig. 8). Tumors from untreated mice showed a decreased expression of CD8 and CD4 and a similar expression of NKp46 compared to preneoplastic mammary glands (Fig. 8). Finally, NKp46, CD8 and CD4 levels were significantly higher in mammary glands of 26-week-old vaccinated mice with respect to the tumor area of untreated 26-week-old mice (Fig. 8).

## Discussion

We found that IL-15 promotes immunosurveillance of mammary carcinogenesis in a transgenic mouse model driven by the activation of HER2/neu oncogene. In addition, we demonstrated that lack of this cytokine caused alterations in immune populations and in vaccine efficacy, showing that IL-15 and/or its regulated populations are necessary for long-term cancer immunoprevention induced by an IL-12-adjuvanted allogeneic HER2/neu-expressing cell vaccine. Lack of IL-15 affected the immune system: transgenic mice deficient for IL-15 showed a lower percentage and a lower number of NK cells (defined as NKp46+DX5+ cells in the lymphocyte gate according to Walzer et al. [19]) and CD8+ T lymphocytes in peripheral blood and in spleen compared to IL-15-proficient mice. Similar alterations in lymphocyte subsets were reported also by Kennedy and co-workers in mice with a different background (C57BL/6 mice [20]) indicating a key role of IL-15 in the regulation of development, survival and differentiation of NK cells and CD8+ T lymphocytes.

IL-15-deficient HER2/neu transgenic mice showed an earlier mammary carcinogenesis, with median latency time of tumor onset 4 weeks before that of IL-15-proficient NeuT mice, suggesting a role for IL-15 in tumor immunosurveillance. A potential role of IL-15 in tumor immunosurveillance has also recently been reported in a mouse model of lymphoma [21]. The above-mentioned immune defects, occurring downstream of IL-15 deficiency, might be at the basis of the decreased immunosurveillance of mammary carcinomas. Indeed NeuT mice knocked out for the perforin gene, which is mainly expressed by NK and CD8+ T lymphocytes, similarly showed a quicker mammary carcinogenesis [22]. A faster onset of mammary carcinomas and a greater tumor multiplicity were found in NeuT mice after depletion of NK cells (by treatment with anti-asialo GM1), but not after depletion of CD8+ cells [22]. Finally, it has been recently demonstrated that the adaptive immune system exerts neither protective nor promoting activities during mammary carcinogenesis and spontaneous metastasis formation in HER2/neu transgenic mice [23]. Taken together, these data point out NK cells as candidate effectors cells of tumor immunosurveillance in this model.

Activities of IL-15 at homeostasis have been extensively studied, whereas its functions during immune activation are not clearly defined. Data presented in this manuscript support a role for IL-15 in cancer immunoprevention. In the absence of IL-15 the preventive vaccine significantly delayed tumor onset but all mice developed tumors with a greater multiplicity. Three phenomena might contribute to the decreased efficacy of the vaccination: (1) quicker mammary carcinogenesis due to the impaired immunosurveillance; (2) absence of direct effects of IL-15; (3) deficit of immune effectors as a consequence of the absence of IL-15. We discuss the three topics below.

In NeuT mice vaccines were more effective when started in preneoplastic stage, corresponding to 6 weeks of age [18]. IL15KO/NeuT mice showed a quicker carcinogenesis and a higher level of HER2/neu oncogene in mammary glands when vaccination started, therefore, this might contribute to decreased vaccine efficacy.

Direct administration of IL-15 has shown antitumor effects in several preclinical mouse tumor models [11]. IL-15 has chemotactic activities and can promote adhesion of leukocytes to vascular endothelial cells [24]. Therefore, in the absence of IL-15, there might be less recruitment of leukocytes in tumor lesions thus decreasing vaccine efficacy. We documented here for the first time that in NeuT mice the preventive vaccine induced a significant decrease in the percentage and in the number of NK cells in peripheral blood and in spleen, and of CD8+ lymphocytes in peripheral blood. A possible explanation is that NK and CD8+ cells are recruited in other tissues (e.g., mammary glands where carcinogenesis takes place) where they can exert immune effector functions. In fact, we showed that NKp46 levels were significantly higher in tumor-free mammary glands from vaccinated mice compared to those of preneoplastic mammary glands from untreated mice, whereas CD8 and CD4 levels were comparable, indicating that the vaccine can induce the recruitment of NK cells in mammary tissue.

Similarly to vaccinated NeuT mice, administration of recombinant human (rh)IL-12 in cancer patients (intravenous [25], intratumoral [26], subcutaneous [27]) induced a dose-dependent lymphopenia starting some hours after the injections and lasting several days. All lymphocyte subsets were affected but NK cells were the most affected ones. It was hypothesized that such lymphopenia derived from the activation of lymphocytes, with their subsequent extravasation and redistribution to tumors and lymphoid organs. Moreover, lymphopenia was also hypothesized to be the result of secondary cytokines produced in response to rhIL-12 [25]. We demonstrated that the IL-12-based vaccine induced the production of IL-15, therefore, IL-15 could be a secondary cytokine cooperating to vaccine efficacy. Interestingly, intravenous administration of rhIL-15 in patients with cancer was also found to induce a rapid reduction (within 40 minutes) in peripheral blood NK cells, apparently due to redistribution, followed by normalization of cell numbers over 3 days. Chronic administration of rhIL-15 subsequently led to an increase in NK cells by hyperproliferation [28]. Simultaneous administration of IL-12 and IL-15 might thus have synergistic effects on NK cells and might be promising to induce antitumor immunity.

Tumors from vaccinated NeuT mice showed several necrotic areas and infiltrating CD3+ lymphocytes, rarely detected in tumors from IL15KO/NeuT mice as well as from untreated NeuT mice. Furthermore, tumors from vaccinated NeuT mice showed few perforin-positive cells, which were completely absent in tumors from IL15KO/NeuT mice. Immunohistochemical analyses previously performed in tumors from NeuT mice (control, IL-12 treated and vaccine treated) showed a huge infiltrate of CD8+ lymphocytes, NK cells, dendritic cells, macrophages and neutrophils in tumors from vaccinated mice supporting this hypothesis [2]. The number of CD8+ lymphocytes, NK cells and dendritic cells increased also in tumors of mice treated with IL-12 alone indicating that recruitment of that type of immune cells is mediated by IL-12 [2].

The efficacy of the preventive vaccine has been demonstrated to rely on the induction of a fast and sustained antibody response against the HER2/neu oncoantigen and on the release of IFN-γ [2, 3, 15, 18]. In IL15KO/NeuT mice the vaccine induced a faster B cell response and a titer of antibody against vaccine cells equal to that induced in IL-15-proficient NeuT mice. Nevertheless, IL15KO/NeuT mice had less IgM and IgG1 anti-vaccine cell antibodies suggesting that antibodies of these isotypes might have a role in long-term cancer immunoprevention. The antibody isotype, which has been mainly associated with the efficacy of different kinds of anti-HER2/neu vaccines, is IgG2a [15, 29–31]. To identify immune mechanisms responsible for the efficacy of the IL-12-adjuvanted allogeneic HER2/neu-expressing cell vaccine, we previously vaccinated NeuT mice knocked out for the Igμ-chain gene (NeuT-μMT). One-third of NeuT-μMT mice was antibody-deficient and did not respond to the vaccine, highlighting the key role of antibodies in cancer immunoprevention in this model. Two-thirds of NeuT-μMT mice, able to bypass the IgM defect, produced high-titer anti-vaccine antibodies. Nevertheless, these mice showed a partial response to the vaccination with a median tumor latency of 45 weeks and this could be related to a lower titer of IgM and IgG1 antibodies against the vaccine compared to NeuT mice [15]. Mouse IgMs can activate the complement [32] while IgG1s can activate antibody-dependent cellular cytotoxicity (ADCC) via FcγRIII and can bind to FcRn involved in the recycling of IgG and in the transport of IgG-bound antigens favoring antigen presentation [33]. The lower production of anti-HER2/neu IgM and IgG1 antibodies found in IL15KO/NeuT mice might thus lead to a decreased activation of complement and antigen presentation resulting in a partial loss of vaccine efficacy. Indeed the complement has been recently shown to be involved in immunosurveillance in NeuT mice [34].

IFN-γ production following in vitro co-culture of splenocytes of vaccinated IL15KO/NeuT mice with tumor cells was comparable to that of vaccinated NeuT mice suggesting that the ability of splenocytes to produce IFN-γ was not affected by the lack of IL-15. Instead, the levels of systemic IFN-γ reached 20 hours after vaccination were lower in IL-15-deficient mice, suggesting that there were less cells that might respond to IL-12 compared to NeuT mice. The most striking difference between IL-15-deficient and IL-15-proficient mice concerned NK cells, CD8+ T lymphocytes and CD8+ memory T cells. To date the induction of a cytotoxic CD8+ T cell response by the preventive vaccine in NeuT mice has been looked for but has never been detected [2, 3, 15, 18]. We suggest that NK cells are necessary to obtain a fully effective immunoprevention of HER2/neu-driven mammary carcinogenesis. On the contrary, Park et al. and Sakai et al. found that vaccine efficacy depends on the induction of anti-HER2/neu antibodies but NK cells and ADCC are dispensable [29, 35]. Such discrepancy could be mainly due to the experimental model used to investigate mechanisms of vaccine efficacy: in fact transplantable tumor models (healthy BALB/c mice challenged with TUBO mammary cancer cells) can elicit profoundly different immune mechanisms than spontaneously arisen carcinogenesis [36, 37]. Other differences concern the different types of vaccines/vaccination protocol. As vaccine, Park et al. used adenoviral vectors expressing the extracellular and transmembrane domains of rat HER2/neu [29]; Sakai et al. used dendritic cells infected with adenoviral vectors expressing the extracellular and transmembrane domains of rat HER2/neu [35] while we used IL-12-adjuvanted vaccine based on allogeneic rat HER2/neu-expressing cells [3].

To date NK cells have been evaluated as tools for cancer immunotherapy (reviewed in [38]). Interestingly, adoptive transfer of syngeneic NK cells preactivated with IL-12/15/18 was effective in decreasing growth of established tumors in mouse models [39] and IL-15 was able to increase efficacy of cetuximab by activating NK cells [40].

In line with our results, it has been recently shown that including IL-15 in HER2/neu-based dendritic cell vaccines and vaccines targeted to professional antigen-presenting cells can increase vaccine efficacy [41, 42]. Moreover, TS/A breast and TRAMP-C2 prostate cancer cells engineered to co-express IL-15 and IL-15Rα showed a lower growth after subcutaneous injection in syngeneic mice and were also effective as a vaccine activating immune responses (mainly mediated by CD8+ and NK cells), which delayed the growth of tumor cell challenges [43].

## Conclusions

Here, by exploiting mice knockout for IL-15, we formally demonstrated for the first time that IL-15 sustains mammary cancer immunosurveillance. In addition, we demonstrated that an IL-12-based cell vaccine can induce IL-15 and that IL-15 is required for the complete efficacy of the vaccine. IL-15 knockout mice had immunological defects in NK and CD8+ cells. These findings increase the understanding of the relationship between the immune system and mammary carcinogenesis, evidencing that mammary tumor onset and long-lasting immunoprevention are controlled by IL-15-mediated immune mechanisms involving in particular NK and CD8+ memory cells. These results support a potential use of IL-15 as an adjuvant in vaccination approaches.

## Additional files

Additional file 1: Figure S1. Concentration of peripheral blood leukocytes, lymphocytes and granulocytes in IL-15-deficient and IL-15-proficient, control and vaccinated NeuT mice. Additional file 2: Figure S2. Concentration of peripheral blood lymphocyte subsets in IL-15-deficient and IL-15-proficient, control and vaccinated NeuT mice. To obtain the number of specific lymphocyte populations, the percentage of B, T, CD4+, CD8+ and NK cells determined by flow cytometry using a lymphocyte gate was multiplied by the number of lymphocyte/ml. Significance of comparisons (Student’s *t* test): ^*^ *p* <0.05, IL15KO/NeuT versus NeuT mice; ^#^ *p* <0.05, vaccinated versus control mice within the same strain. Mean ± SEM is shown (three to six mice per group). Additional file 3: Figure S3. Spleen cell yield and splenocyte subset yield of IL-15-deficient and IL-15-proficient, control and vaccinated, NeuT mice. To obtain the absolute number of subsets of splenocytes, the percentage of B, T, CD4+, CD8+ and NK cells determined by flow cytometry was multiplied by the total number of splenocytes. Significance of comparisons (Student’s *t* test): ^*^ *p* <0.05, IL15KO/NeuT versus NeuT mice; ^#^ *p* <0.05, vaccinated versus control mice within the same strain; ^§^ *p* <0.05, 15 versus 42-week-old mice within the same strain. Mean ± SEM is shown (three to six mice per group). Additional file 4: Figure S4. Systemic cytokines induced by the preventive vaccine in IL-15-deficient and IL-15-proficient NeuT mice. Analyses were performed 20 hours after the administration of the vaccine at the end of the third vaccination cycle (15-week-old mice). Mean ± SEM is shown (five mice per group). Significance of comparisons (Wilcoxon nonparametric test): ^*^ *p* <0.05, IL15KO/NeuT versus NeuT mice; ^#^ *p* <0.05, vaccinated versus control mice within the same strain. Production of IL-3 and IL-6 was not detected.

## Abbreviations

ADCC: antibody-dependent cellular cytotoxicity
ELISA: enzyme-linked immunosorbent assay
FITC: fluorescein isothiocyanate
G(M)-CSF: granulocyte (macrophage)-colony stimulating factor
H&E: hematoxylin and eosin
IFN-γ: interferon-gamma
IL-1α: interleukin-1 alpha
IL-2: interleukin-2
IL-5: interleukin-5
IL-13: interleukin-13
IL-12: interleukin-12
IL-15: interleukin-15
IL15KO/NeuT: BALB/c mice transgenic for rat HER2/neu oncogene and knockout for IL-15
IL-17: interleukin-17
KC: keratinocyte chemoattractant
NeuT: BALB/c mice transgenic for rat HER2/neu oncogene
Ig: immunoglobulin
MCP-1: monocyte chemoattractant protein-1
MIP-1α: macrophage inflammatory protein-1 alpha
NK: natural killer
PBS: phosphate-buffered saline
PE: phycoerythrin
RANTES: regulated on activation, normal T cell expressed and secreted
rh: recombinant human
SEM: standard error of the mean
