# Supplementary figures and images for: Interleukin-15 is required for immunosurveillance and immunoprevention of HER2/neu-driven mammary carcinogenesis
Source: Breast Cancer Res. 2015 May 22;17(1):70. doi: 10.1186/s13058-015-0588-x (PMC4462012; doi:10.1186/s13058-015-0588-x)

Figure S1

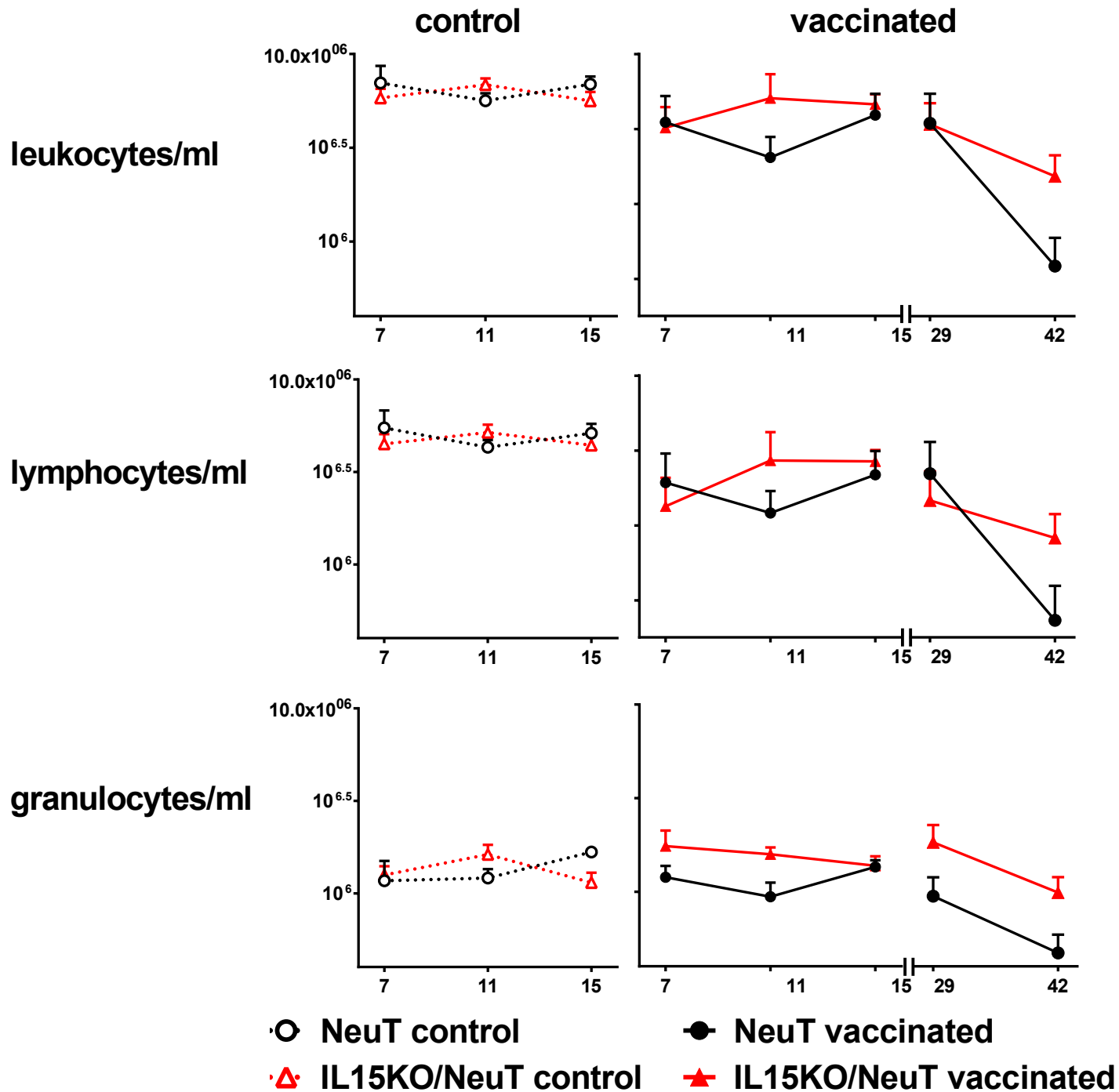

Supplement: Additional file 1: Figure S1. — Concentration of peripheral blood leukocytes, lymphocytes and granulocytes in IL-15-deficient and IL-15-proficient, control and vaccinated NeuT mice. [file 13058_2015_588_MOESM1_ESM.pdf]

# Figure S2

**NK**

**CD8+**

**CD4+**

**T**

**B**

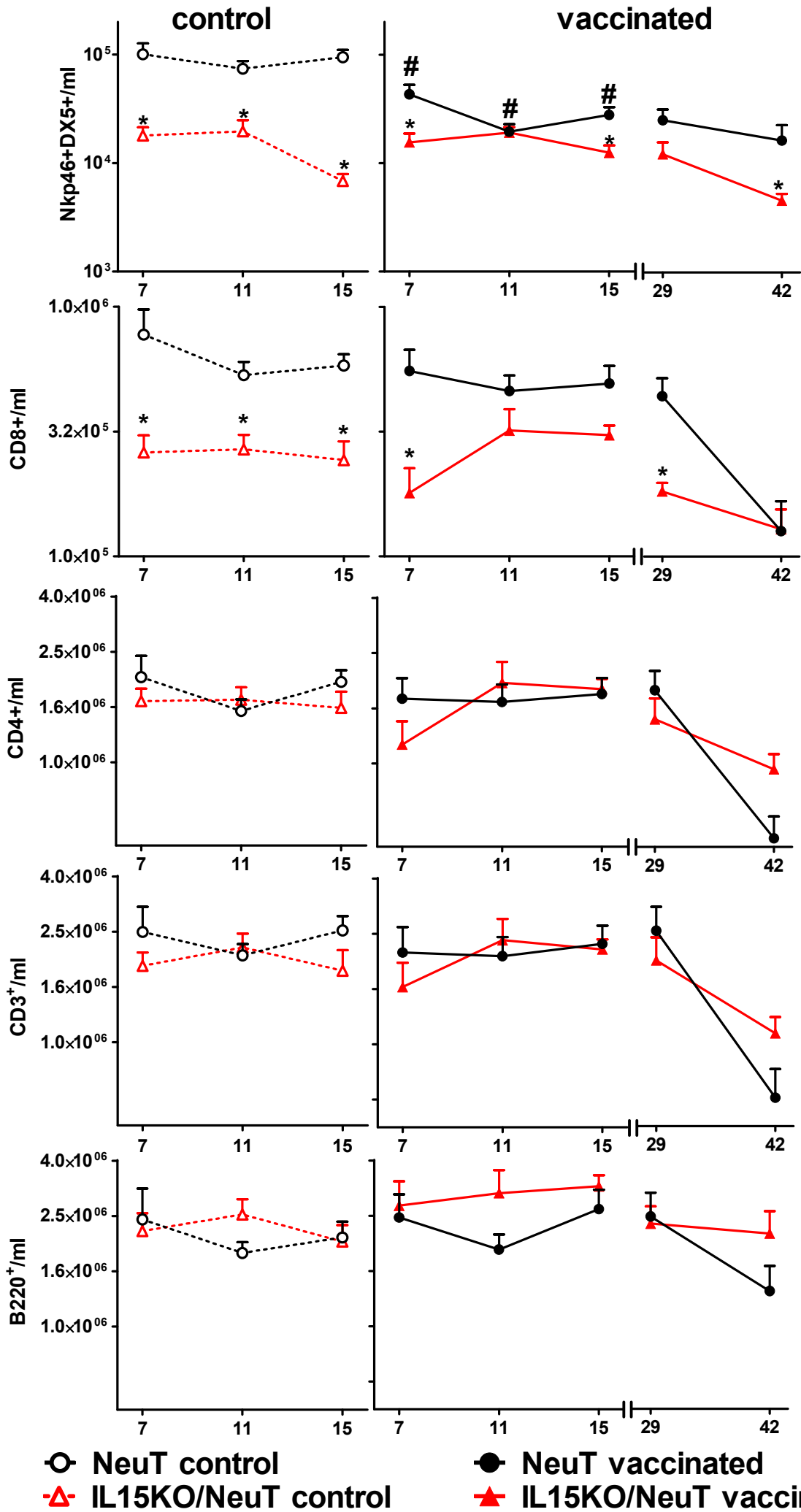

Supplement: Additional file 2: Figure S2. — Concentration of peripheral blood lymphocyte subsets in IL-15-deficient and IL-15-proficient, control and vaccinated NeuT mice. To obtain the number of specific lymphocyte populations, the percentage of B, T, CD4+, CD8+ and NK cells determined by flow cytometry using a lymphocyte gate was multiplied by the number of lymphocyte/ml. Significance of comparisons (Student’s t test): * p <0.05, IL15KO/NeuT versus NeuT mice; # p <0.05, vaccinated versus control mice within the same strain. Mean ± SEM is shown (three to six mice per group). [file 13058_2015_588_MOESM2_ESM.pdf]

**Figure S3**

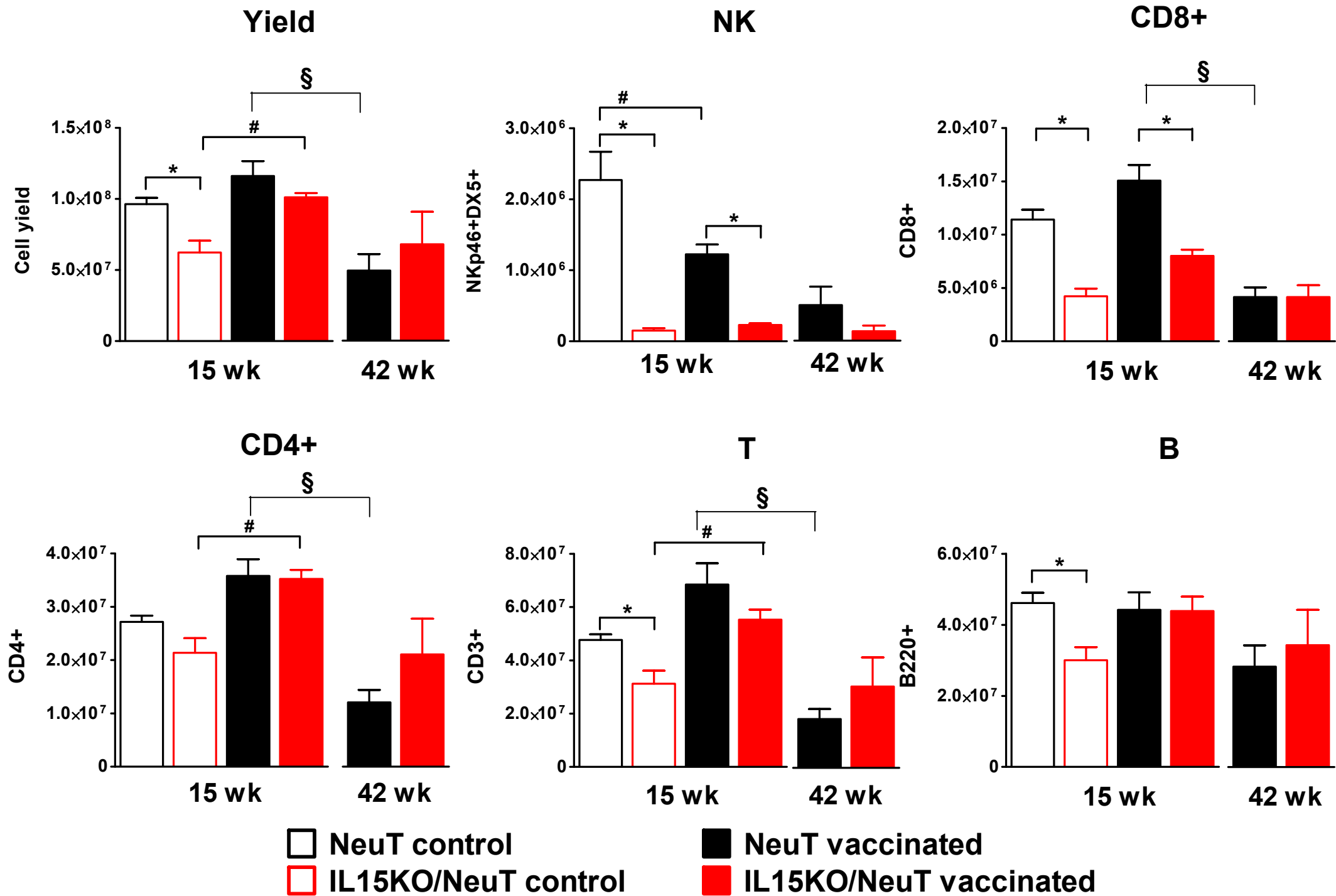

Supplement: Additional file 3: Figure S3. — Spleen cell yield and splenocyte subset yield of IL-15-deficient and IL-15-proficient, control and vaccinated, NeuT mice. To obtain the absolute number of subsets of splenocytes, the percentage of B, T, CD4+, CD8+ and NK cells determined by flow cytometry was multiplied by the total number of splenocytes. Significance of comparisons (Student’s t test): * p <0.05, IL15KO/NeuT versus NeuT mice; # p <0.05, vaccinated versus control mice within the same strain; § p <0.05, 15 versus 42-week-old mice within the same strain. Mean ± SEM is shown (three to six mice per group). [file 13058_2015_588_MOESM3_ESM.pdf]

Figure S4

□ NeuT control      ■ NeuT vaccinated  
□ IL15KO/NeuT control      ■ IL15KO/NeuT vaccinated

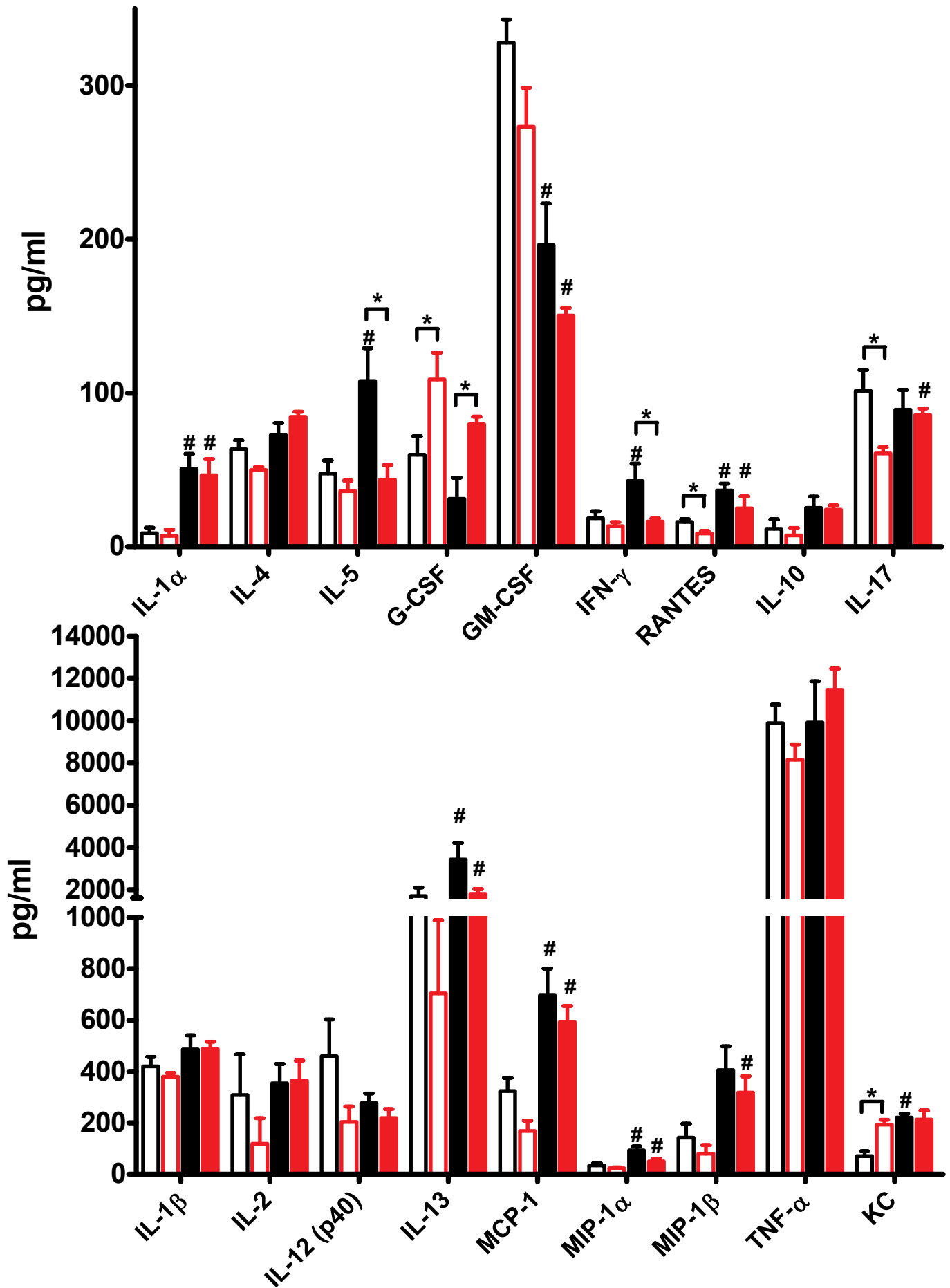

Supplement: Additional file 4: Figure S4. — Systemic cytokines induced by the preventive vaccine in IL-15-deficient and IL-15-proficient NeuT mice. Analyses were performed 20 hours after the administration of the vaccine at the end of the third vaccination cycle (15-week-old mice). Mean ± SEM is shown (five mice per group). Significance of comparisons (Wilcoxon nonparametric test): * p <0.05, IL15KO/NeuT versus NeuT mice; # p <0.05, vaccinated versus control mice within the same strain. Production of IL-3 and IL-6 was not detected. [file 13058_2015_588_MOESM4_ESM.pdf]
